# Supplementary material for: Human leukocyte antigen-DQA1*04:01 and rs2040406 variants are associated with elevated risk of childhood Burkitt lymphoma
Source: Commun Biol. 2024 Jan 5;7:41. doi: 10.1038/s42003-023-05701-5 (PMC10770398; doi:10.1038/s42003-023-05701-5)
Supplement: Supplementary file 2 — Supplementary Information [file 42003_2023_5701_MOESM2_ESM.pdf]

**Human Leukocyte Antigen-*DQA1*04:01* and rs2040406 Variants Are Associated with Elevated Risk of Childhood Burkitt Lymphoma**

**Liu et al.**

**Supplementary Information**

# Contents of Supplementary Tables

**Contents**


| Table name | Title | Key methods/results | Page number |
|---|---|---|---|
| Supplementary Table 1 | Table showing number of imputed HLA variants according to mean allele frequency and dosage $R^2$ of genotyping and imputation | The table shows the number of variants excluded and included in the analysis. | 14 |
| Supplementary Table 2 | List of 12 HLA alleles a priori-specified because of previous associations with malaria, Epstein-Barr virus (EBV), or Burkitt lymphoma (BL) | The table shows a list of previously reported 12 HLA alleles that were associated with malaria, EBV, or BL. These HLA alleles were considered as *a priori*-specified in the analysis. | 15 |
| Supplementary Table 3 | Associations of Burkitt lymphoma (BL) with nine HLA variants with a priori-specified associations with malaria, Epstein-Barr virus (EBV), or BL in EMBLEM and Malawi | The table shows associations of BL with the nine a priori-specified HLA alleles based on the generalized linear mixed models. Only one HLA allele showed suggestive associations with BL in the analysis (*HLA-A*23:01*). | 16 |
| Supplementary Table 4 | Associations of Burkitt lymphoma (BL) with three a priori-specified HLA alleles in EMBLEM Uganda | The table shows associations of BL with the three a priori-specified HLA alleles in EMBLEM Uganda population based on the generalized linear mixed models. Two HLA alleles showed suggestive associations with BL in EMBLEM Uganda (*HLA-A*02*, and *-B*41*). | 17 |
| Supplementary Table 5 | Top HLA alleles associated with Burkitt lymphoma (BL) in EMBLEM and Malawi | The table shows results of the top HLA alleles associated with BL based on the generalized linear mixed models. However, these HLA alleles were not significantly associated with BL conditioning on *HLA-DQA1*04:01*. | 18 |
| Supplementary Table 6 | Country-specific associations with Burkitt lymphoma (BL) with identified top HLA allele, Haplotype, GWAS loci | The table shows results of the country-specific associations with Burkitt lymphoma with identified top HLA allele, Haplotype, GWAS loci based on the generalized linear mixed models. No heterogeneity of associations across countries were observed for those top BL-HLA variants. | 19 |
| Supplementary Table 7 | Sensitivity analyses 2-5 of associations of Burkitt lymphoma (BL) with HLA-DQA1*04:01 in EMBLEM and Malawi studies | The table shows results based on our sensitivity analyses 2-5, which indicates a robustness of our findings. | 20 |

| | | | |
|---|---|---|---|
| Supplementary Table 8 | Association of Burkitt lymphoma (BL) with top GWAS loci (*P*<$1.1\times10^{-6}$) in the HLA region in EBMLEM and Malawi studies | The table shows results of the top SNPs associated with BL based on the generalized linear mixed models and their annotations obtained from ANNOVAR. However, these SNPs were not significantly associated with BL conditioning on SNP rs2040406 | 21 |
| Supplementary Table 9 | Association of Burkitt lymphoma (BL) with top loci with previously reported pleiotropic associations with anti-EBV IgG | The table shows association of Burkitt lymphoma with top loci with previously reported pleiotropic associations with anti-EBV IgG levels based on the generalized linear mixed models. We have confirmed our hypothesis by showing that those anti-EBV IgG-SNPs have suggestive evidence of associations with BL in the analysis. | 22 |
| Supplementary Table 10 | Table showing functional annotation for locus rs2040406 using several public databases | The table summarizes the evidence of BL-SNP rs2040406 in EBV control. | 23 |
| Supplementary Table 11 | Association between Burkitt lymphoma (BL) and common HLA alleles (AF>5%; reference: rare alleles) in EMBLEM and Malawi. | The table shows association of Burkitt lymphoma with common HLA alleles based on the generalized linear mixed models. We hypothesized that BL might be associated with common versus rare HLA alleles, but this hypothesis was not supported by the results. | 24 |
| Supplementary Table 12 | Association between Burkitt lymphoma (BL) and HLA zygosity in EMBLEM and Malawi | The table shows association of Burkitt lymphoma with HLA zygosity based on the generalized linear mixed models. We hypothesized that BL might be associated with HLA zygosity, but this hypothesis was not supported by the results. | 25 |

# Supplementary Figures

**Supplementary Figure 1**. Comparison of imputed HLA alleles with two-field sequencing-based HLA typing, as a gold standard, in a subset of 600 participants in the dataset with paired data. A) The imputation accuracy for seven classical HLA genes at two-field resolution (allele frequency >1%). B) The correlation between imputed at two-field resolution using the multi-ancestry HLA reference panel versus SBT-typed dosages of common classical HLA alleles (allele frequency >1%).

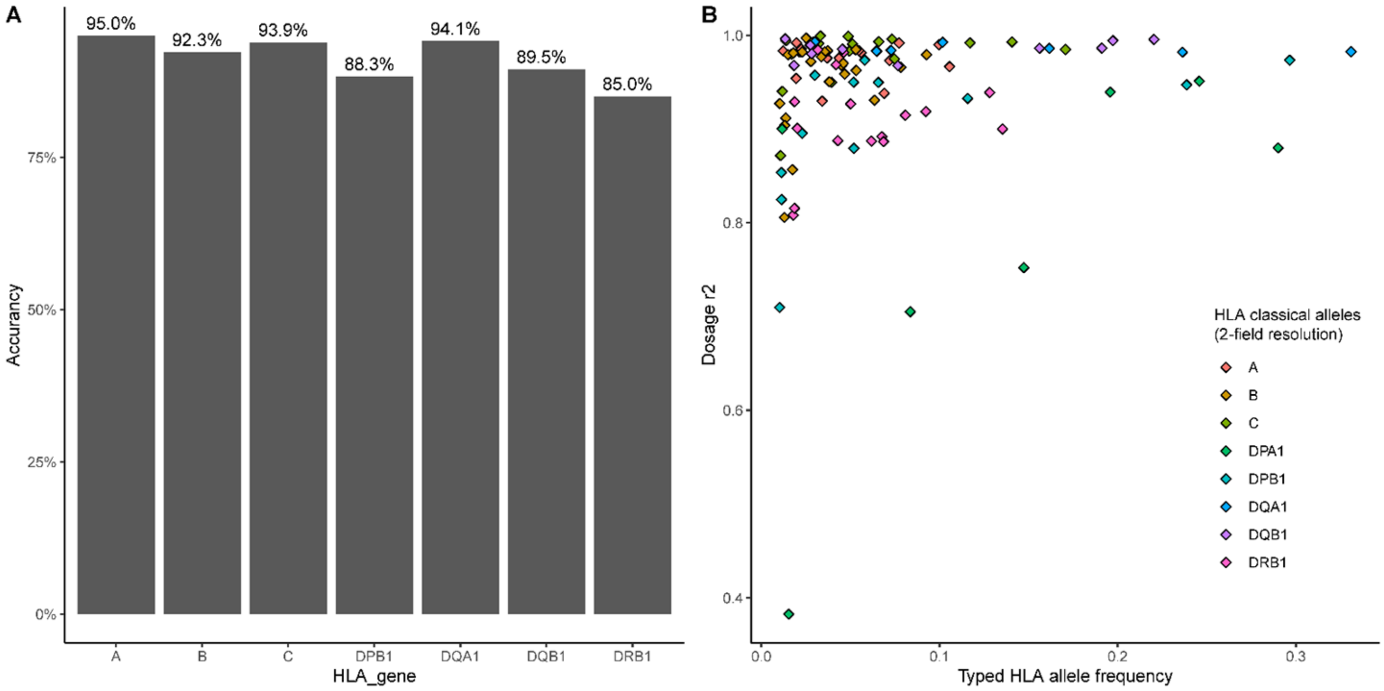

**Supplementary Figure 2.** Plots of the first two country-specific principal component (PC) based on 800 Burkitt lymphoma cases and 3,845 controls. Principal component 1 (PC1) is displayed on the x-axis, and principal component 2 (PC2) is displayed on the y-axis.

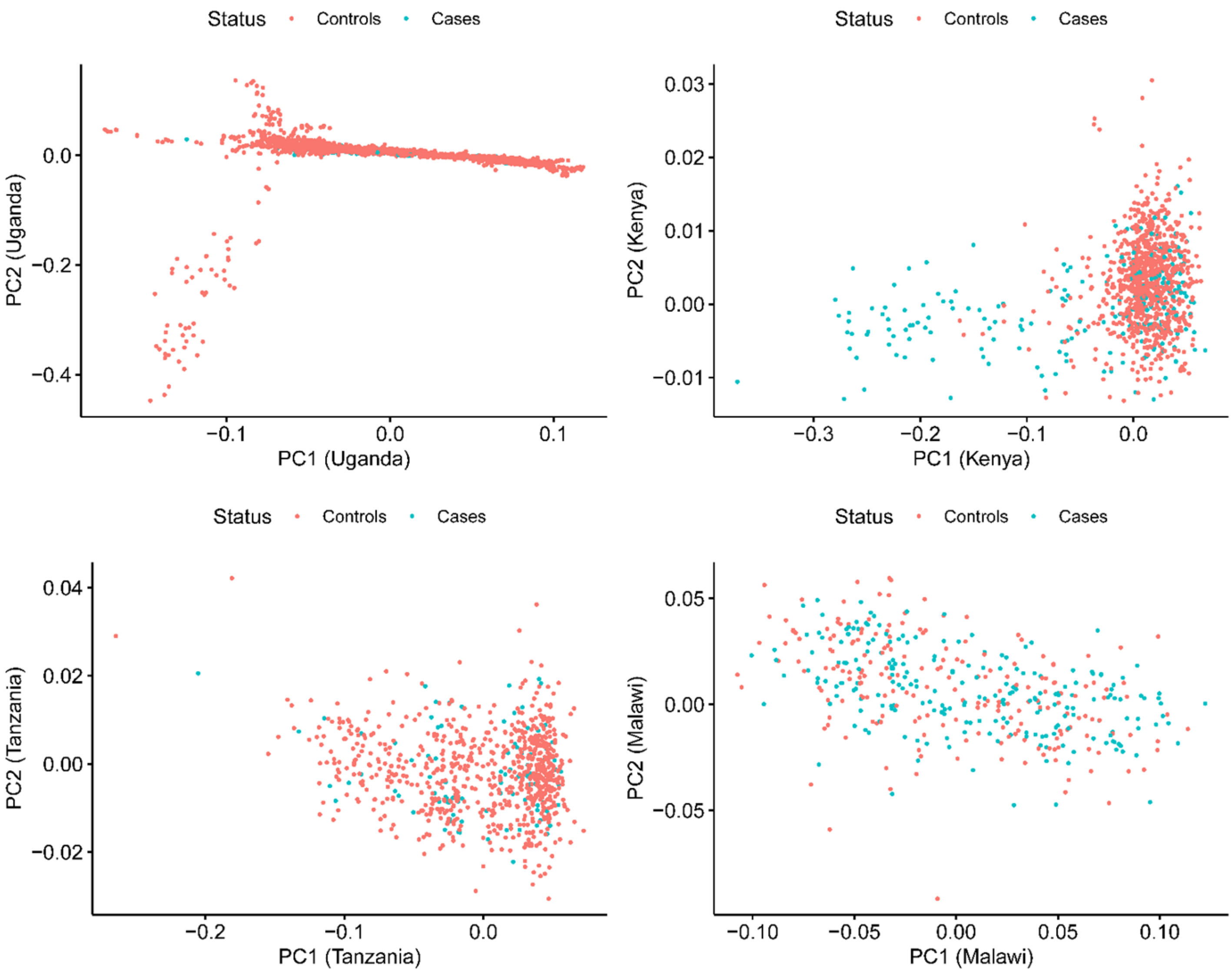

**Supplementary Figure 3.** Region association plots of variants in the major histocompatibility complex region and Burkitt lymphoma from unconditional analysis without an adjustment for *P. falciparum* positivity (800 Burkitt lymphoma cases and 3,845 controls). The adjusted odds ratios (ORs) and p values for the top SNP rs2040406 and HLA allele DQA1*04:01 are 1.42 ($P$=6.51×10$^{-8}$) and 1.54 ($P$=1.77×10$^{-5}$), respectively. All statistical tests are 2-sided.

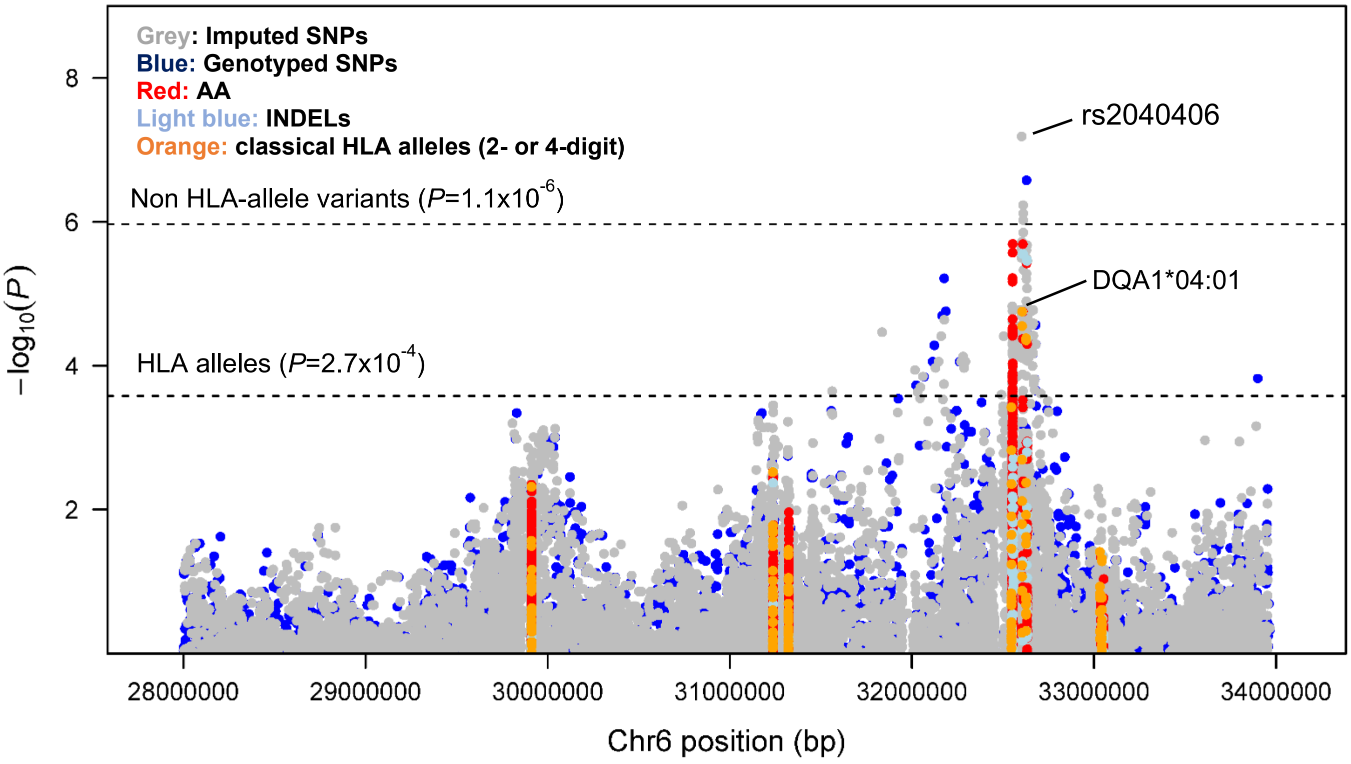

**Supplementary Figure 4.** Plots showing Haplotype structure of HLA alleles for Burkitt lymphoma (BL) cases and controls of East African ancestry (800 BL cases and 3,845 controls) and Ghana (968 individuals). The vertical stacked bar indicates each of the seven HLA genes, and the queues of the bars correspond to their physical order in the HLA region. A tile of a bar indicates an HLA allele, and a segment connects 2 alleles on adjacent genes. The height of the tile and the thickness of the segment correspond to the allele frequency of the HLA allele and haplotype frequency between the 2 HLA alleles, respectively. The frequency of the common haplotype DRB1*03:03-DQB1*04:02-DQA1*04:01 is increased in cases compared with population-based controls of East African. The haplotype DRB1*08:04-DQB1*03:01-DQA1*04:01 is observed in Ghana population but not in East African.

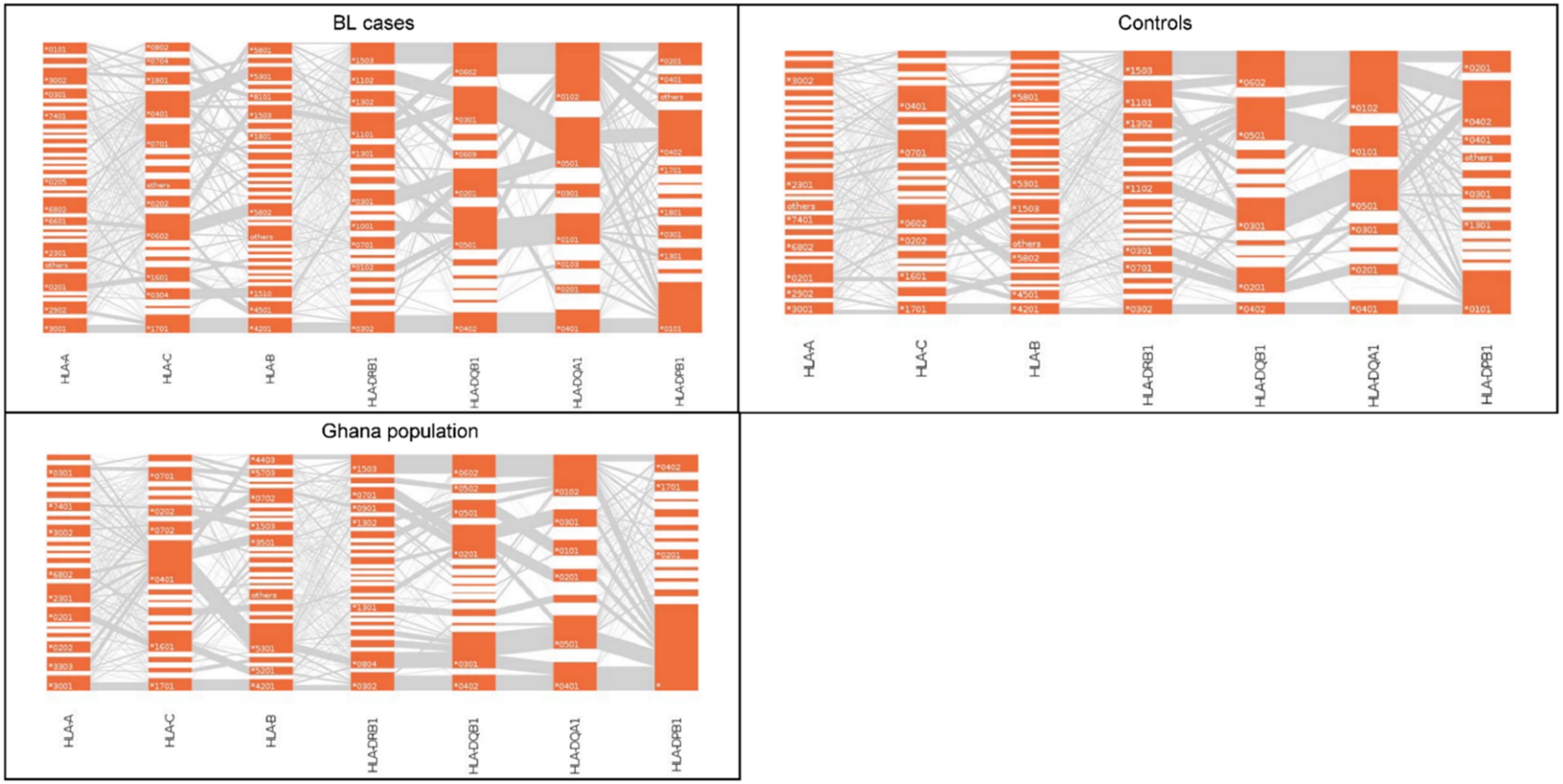

**Supplementary Figure 5.** Regional meta-analyses association plot of HLA-DQA1 and -DQB1 including the rs2040406 that passed the genome-wide significance threshold, showing genes located at or near the significant loci, the extent of LD within the selected region in 1000 genome in AFR population (http://locuszoom.org/). Regional meta-analyses GWAS statistics obtained from GLMM models assuming an additive genetic model with sex, age, *falciparum* positivity, principal components, and relatedness as covariates, summarizing effect estimates across four East African countries.

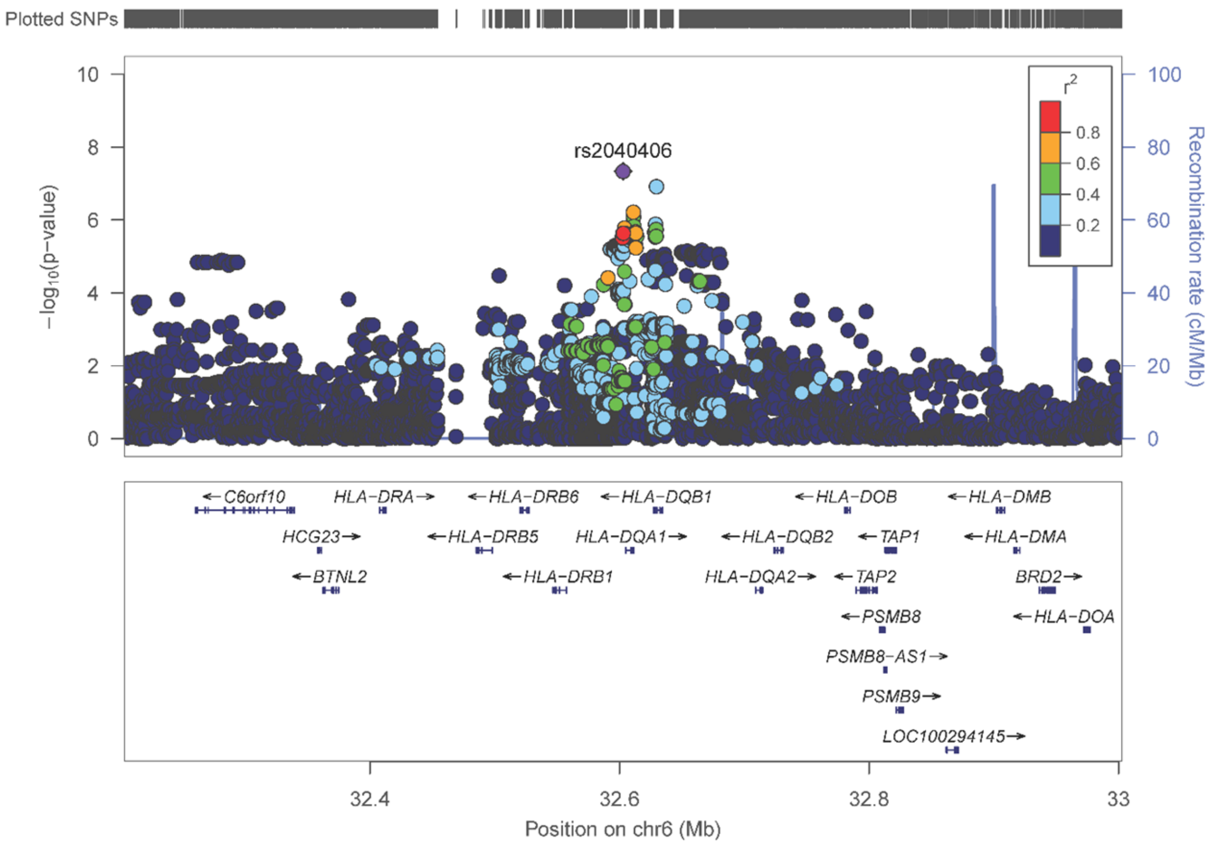

**Supplementary Figure 6.** Three-dimensional ribbon models for the HLA-DQA1 (green) and HLA-DQB1 (purple) proteins. This structure is based on Protein Data Bank entry 4d8p, with a direct view of the peptide-binding groove. The figure highlights amino acid in position 53 that was associated with BL in those carrying glutamine versus those carrying lysine or arginine in unconditional association analyses. All statistical tests are 2-sided.

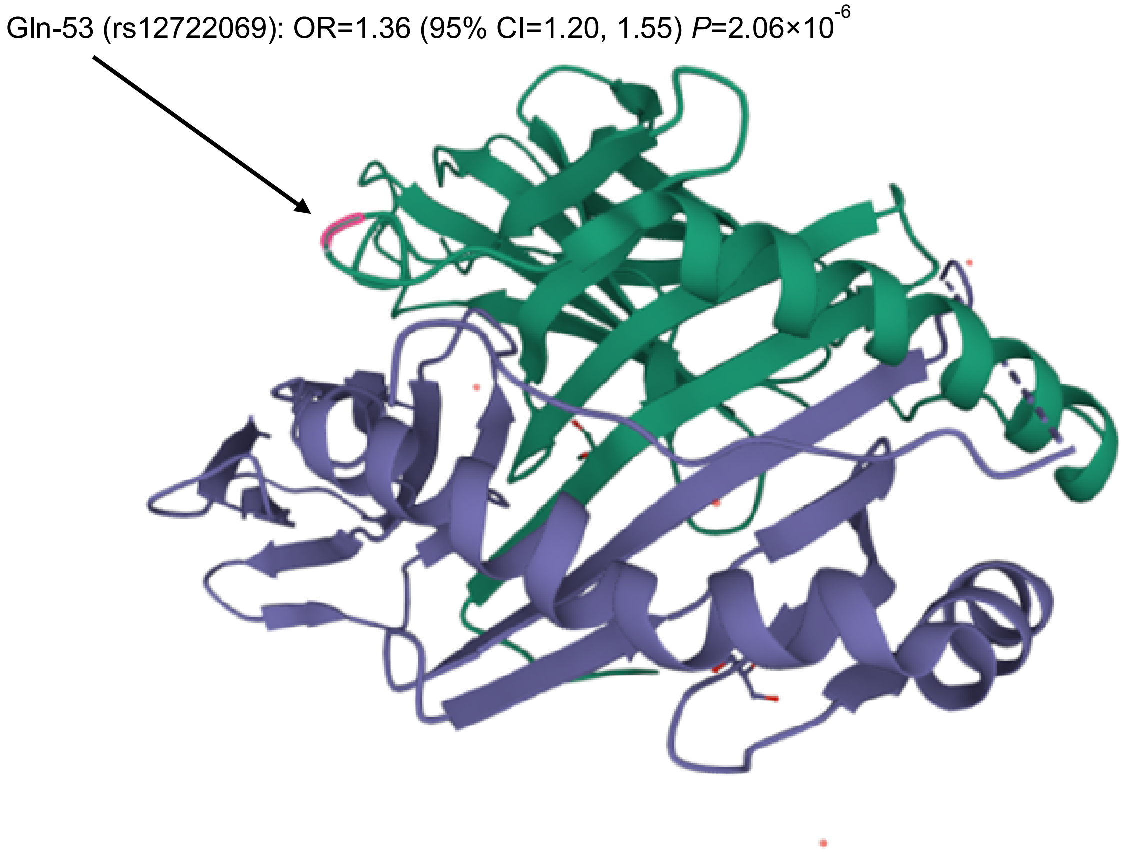

**Supplementary Figure 7.** Boxplots showing *P. falciparum* parasite density according to genotypes for locus rs2040406 and HLA*DQA1*04:01 alleles (top hits for BL) among 3,845 controls. Results are shown separately for EMBLEM (Uganda, Kenya, and Tanzania) and for Malawi. The box plot shows the interquartile range (IQR) between the first (Q1) and third (Q3) quartiles at the ends and the median as a central line in the box. Actual values are plotted as dots within the box plot. All statistical tests are 2-sided.

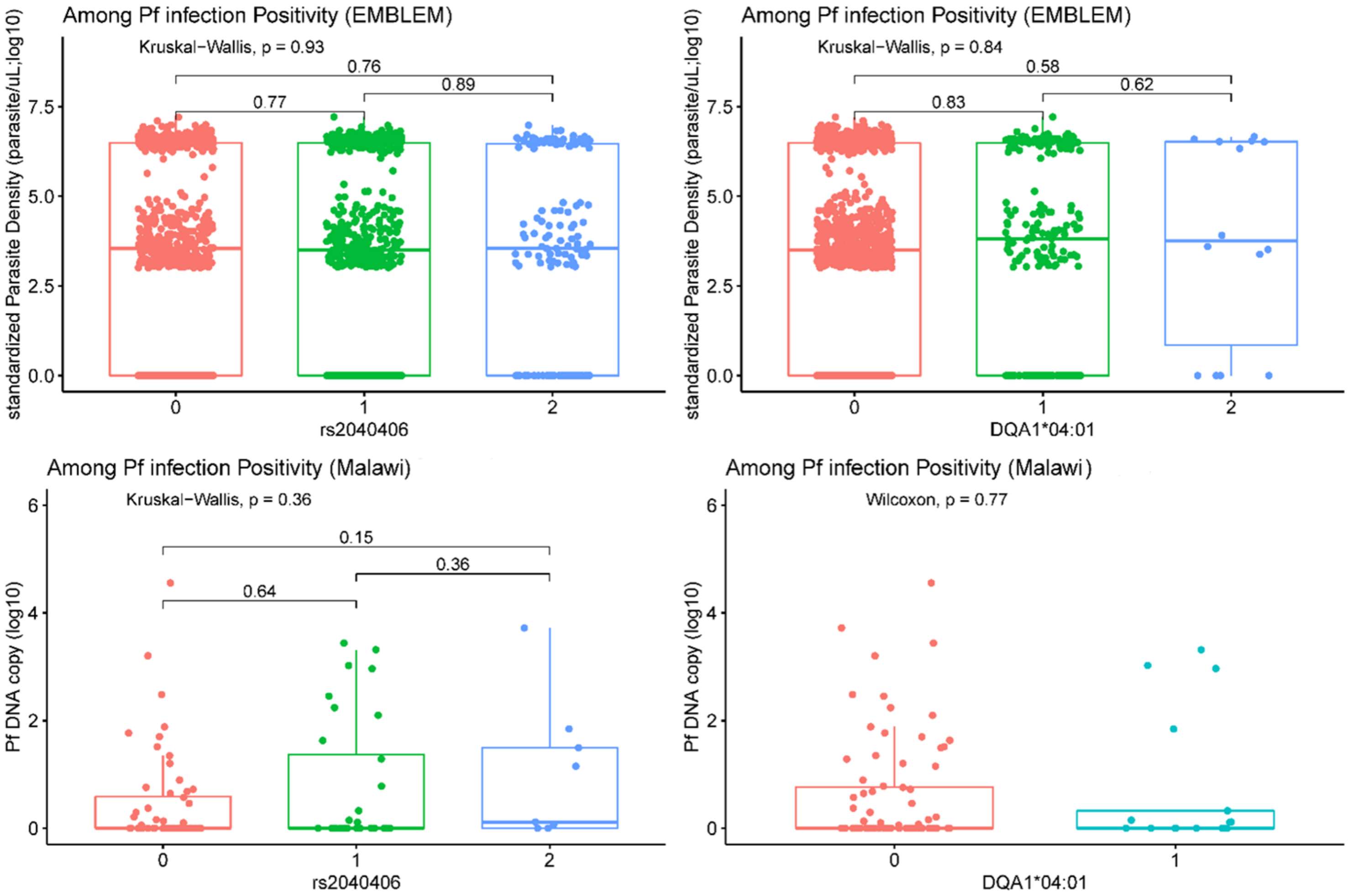

**Supplementary Figure 8.** Regional association plots of variants in the major histocompatibility complex region and *P. falciparum* infection in EMBLEM and Malawi study (sensitivity analysis 1) based on 1857 children who are positive for *Plasmodium falciparum* and 1943 who are negative. All statistical tests are 2-sided.

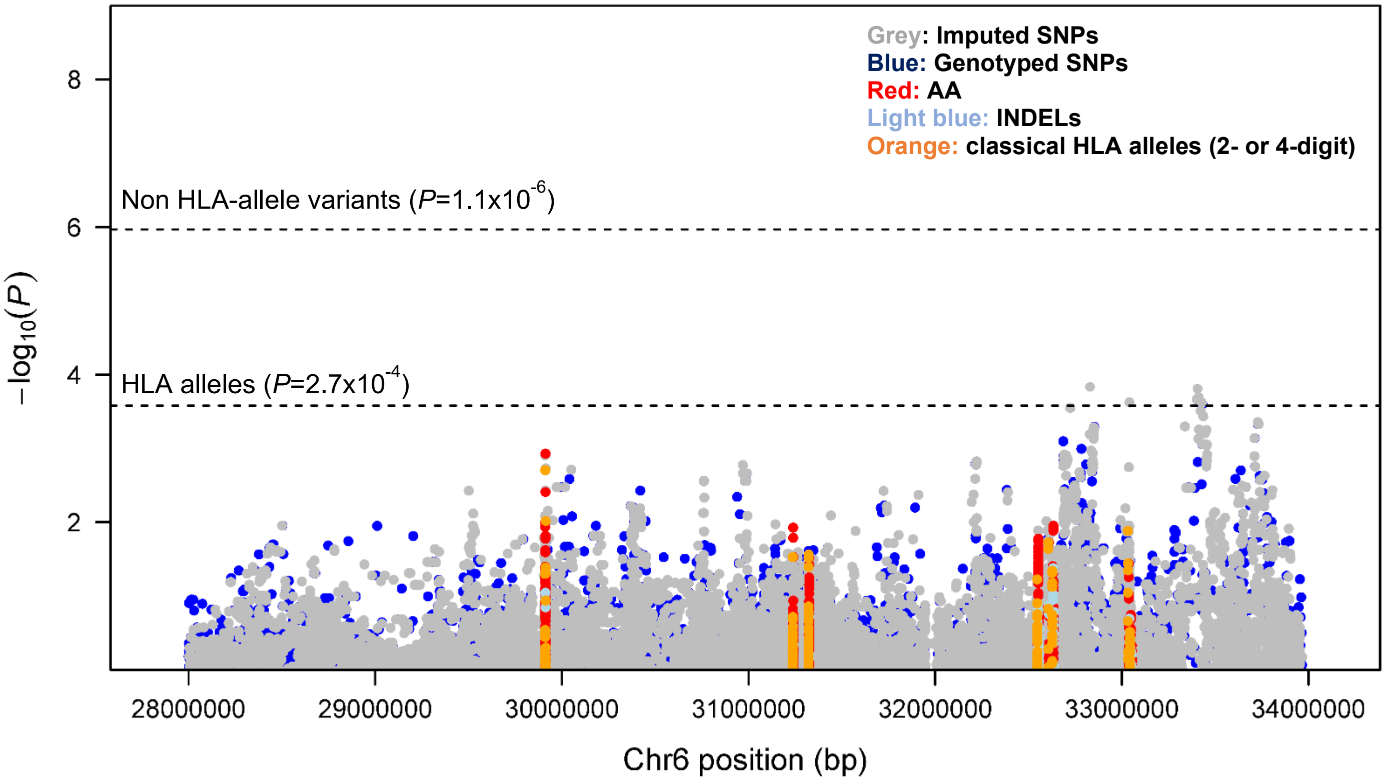

**Supplementary Figure 9.** eQTLs in EBV transformed lymphocytes for rs2040406 (obtained from GTEs portal (https://gtexportal.org/home/; assessed on January 24, 2023). The G allele is associated with risk for BL. The ends of the boxes indicate the first quartile (Q1) and third (Q3) quartiles of the interquartile range (IQR), while the white line in the middle indicates the median result. All statistical tests are 2-sided.

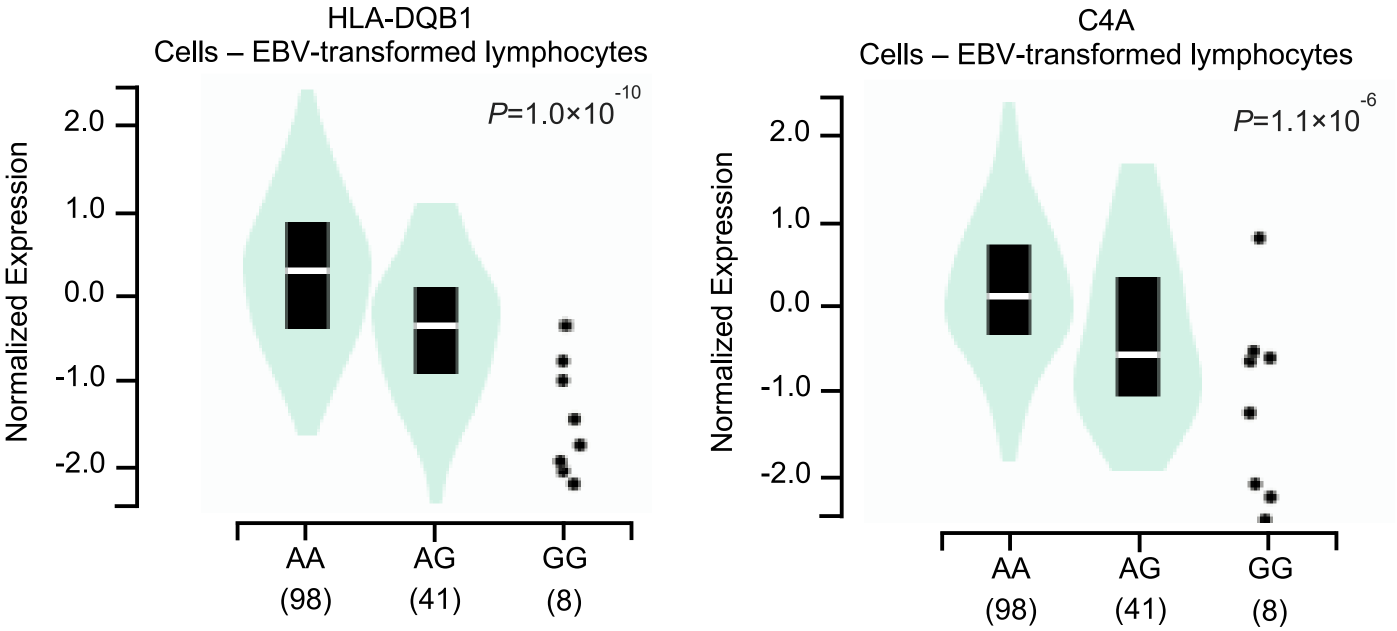

**Supplementary Figure 10.** Flow chart of inclusion and exclusion of HLA variants

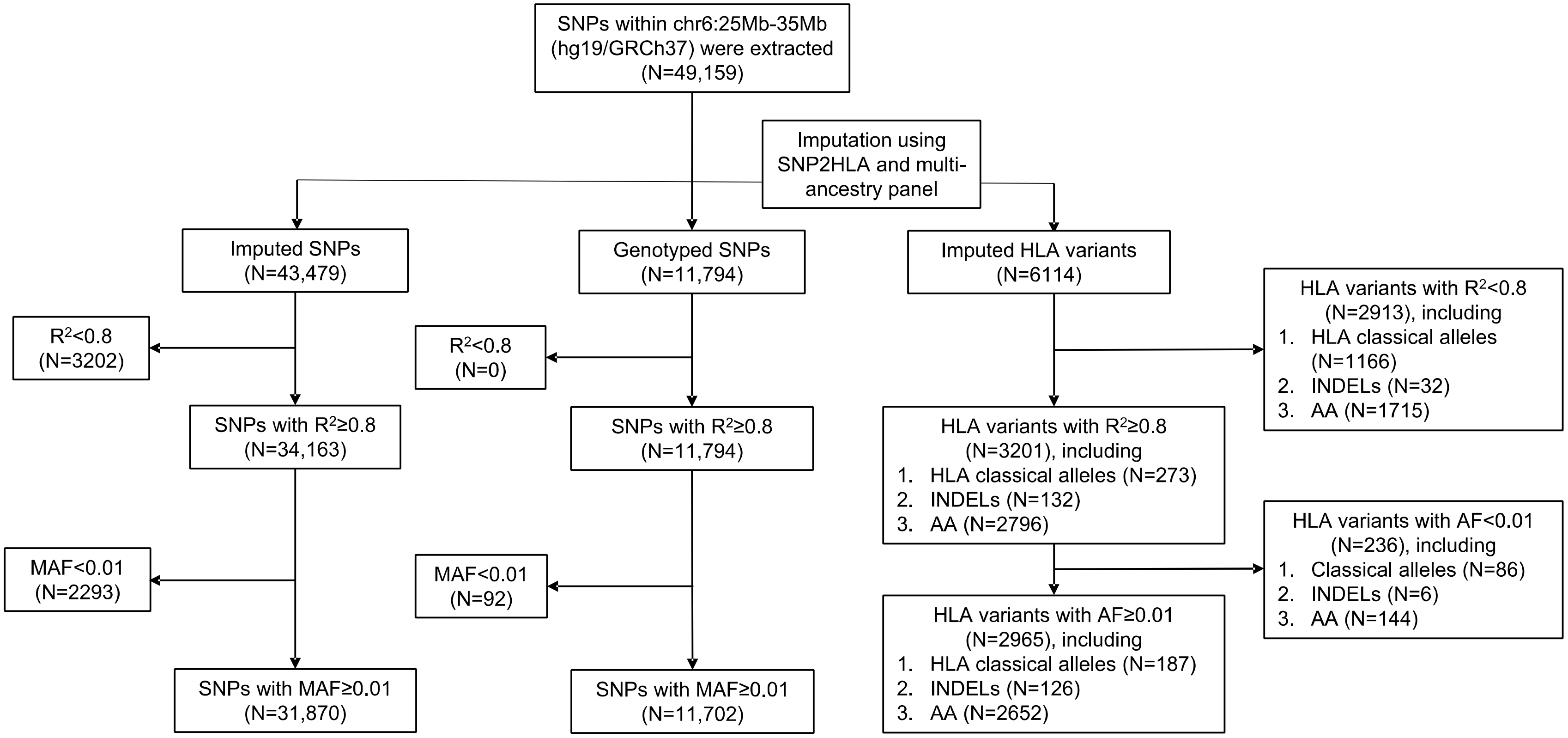

**Supplementary Figure 11.** Stacked bar graphs showing HLA allele frequencies (2-field resolution) by country. The relative height of the box indicates the frequency of the HLA allele. Some alleles in with a frequency >5% are shown, while those with a lower frequency are grouped together under "other". The alleles with notable differences in allele frequencies between Ghana and East Africa are boxed to highlight these patterns, e.g., HLA-B*53:01 (HLA-B*53:01 is equivalent to B*53 since there were no 4-digit HLA alleles other than B*53:01 in -B*53) and -DQA1*04:01.

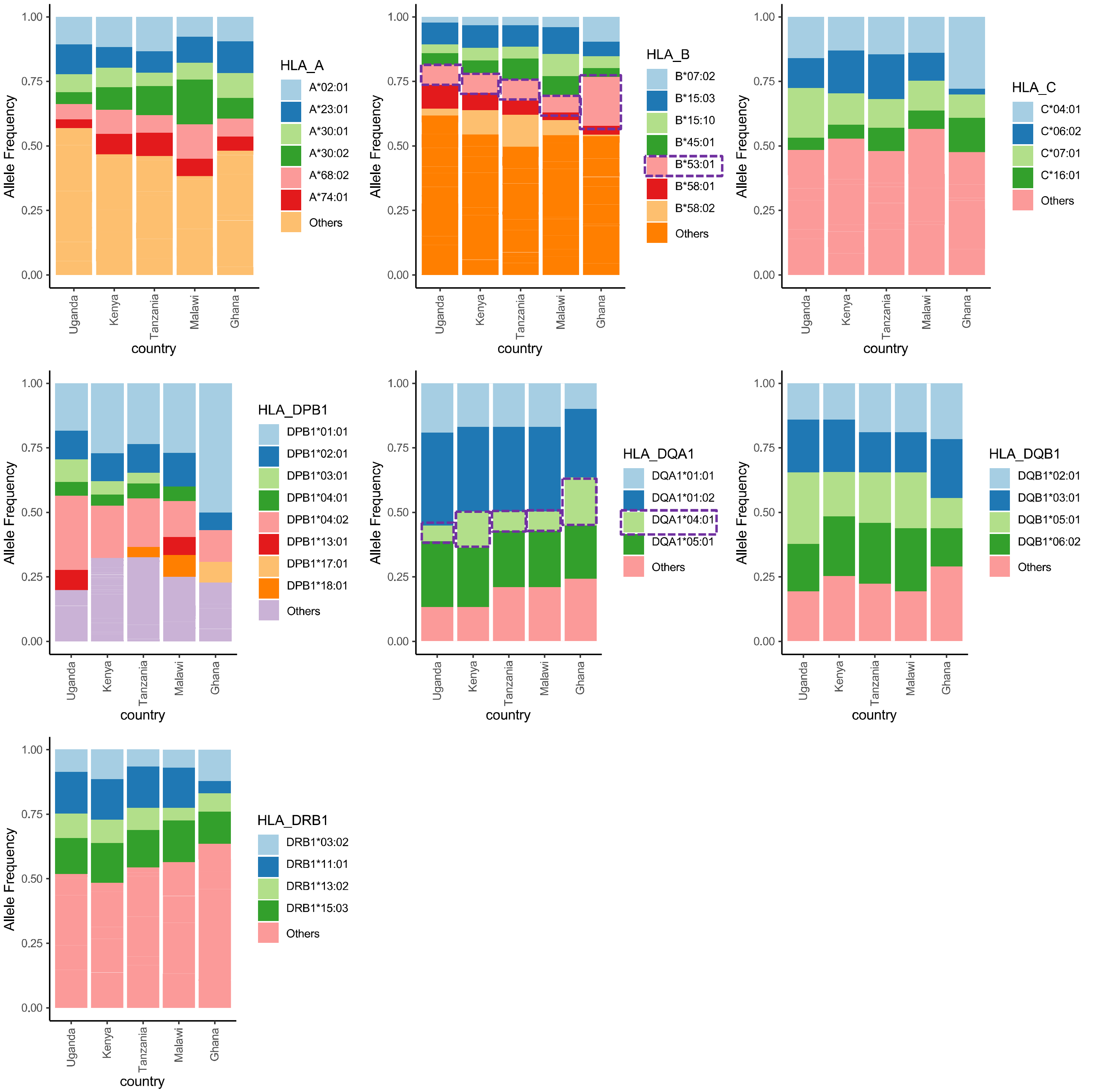

**Supplementary Table 1.** Table showing number of imputed HLA variants according to mean allele frequency and dosage $R^2$ of genotyping and imputation

| HLA variants | $R^2$ <0.8 (n=6115) | | $R^2$ ≥0.8 (n=49,158) | | Total |
|---|---|---|---|---|---|
| | MAF <0.01 | MAF ≥0.01 | MAF <0.01 | MAF ≥0.01 | |
| **SNPs (n)** | 2132 | 1070 | 2385 | 43,572 | 49,159 |
| **Imputed HLA variants (n)** | | | | | |
| **Classical alleles (n)** | 1160 | 6 | 86 | 187 | 1439 |
| **INDELs (n)** | 21 | 11 | 6 | 126 | 164 |
| **Amino acid polymorphisms (n)** | 1690 | 25 | 144 | 2652 | 4511 |
| **Total** | 5003 | 1112 | 2621 | 46,537 | 55,273 |

**Supplementary Table 2.** List of 12 HLA alleles *a priori*-specified because of previous associations with malaria, Epstein-Barr virus (EBV), or Burkitt lymphoma (BL)

| Reported HLA alleles | First author and journal | Year of Publication | Country | Region in Africa |
|---|---|---|---|---|
| **Severe Malaria** | | | | |
| B*53 | Hill et al., Nature | 1991 | Gambia | West |
| DRB1*1302 | Hill et al., Nature | 1991 | Gambia | West |
| DQB1*0501 | Hill et al., Nature | 1991 | Gambia | West |
| DRB1*04 | Osafo-Addo et al, Am J Trop Med Hyg | 2008 | Northern Ghana | West |
| A*30:01 | Lyke et al, Tissue Antigens | 2011 | Mali | West |
| A*33:01 | Lyke et al, Tissue Antigens | 2011 | Mali | West |
| **Anti-EBV antibodies** | | | | |
| DRB1*1501 | Sallah et al, Nat Comm | 2020 | Kyamulibwa, Uganda | East |
| A*23:01 | Sallah et al, Nat Comm | 2020 | Kyamulibwa, Uganda | East |
| **BL**[a] | | | | |
| A*29 | Hall et al, Hum Immunol | 1982 | Tanzania, Kenya | East |
| A*02 | Kirimunda et al, Br J Haematol | 2020 | Northern Uganda | East |
| B*41 | Kirimunda et al, Br J Haematol | 2020 | Northern Uganda | East |
| B*58 | Kirimunda et al, Br J Haematol | 2020 | Northern Uganda | East |

[a] Studies with a sample size of > 100 were included.

**Supplementary Table 3.** Associations of Burkitt lymphoma (BL) with nine HLA variants with *a priori*-specified associations with malaria, Epstein-Barr virus (EBV), or BL in EMBLEM and Malawi

| HLA alleles [a] | Frequency | | Meta-analysis [b] | | | |
|---|---|---|---|---|---|---|
| | Controls | Cases | OR | *P* | $P_{het}$ [c] | $I^2$ |
| B*53 | 0.08 | 0.07 | 0.98 | 0.847 | 0.474 | 0.0 |
| DRB1*1302 | 0.09 | 0.08 | 0.89 | 0.305 | 0.215 | 32.8 |
| DQB1*0501 | 0.25 | 0.22 | 0.92 | 0.287 | 0.580 | 0.0 |
| A*30:01 | 0.07 | 0.08 | 1.12 | 0.374 | 0.142 | 45.0 |
| A*23:01 | 0.10 | 0.08 | 0.77 | 0.027 | 0.673 | 0.0 |
| A*29 | 0.07 | 0.07 | 1.16 | 0.200 | 0.424 | 0.0 |
| A*02 | 0.19 | 0.16 | 0.92 | 0.326 | 0.012 | 72.6 |
| B*41 | 0.02 | 0.02 | 0.89 | 0.656 | 0.013 | 72.2 |
| B*58 | 0.14 | 0.13 | 0.95 | 0.561 | 0.103 | 51.5 |

[a] HLA alleles DRB1*04, A*33:01 and DRB1*1501 were excluded due to low frequency or imputation quality.

[b] Estimates were obtained from generalized linear mixed models (GLMMs), accounting for genetic relatedness and adjusting for sex, age (continuous), *Plasmodium falciparum* positivity, and population structure using the top 3 principal components (PCs). Association statistics were conducted by a fixed-effect meta-analysis across four countries. All statistical tests are 2-sided.

[c] Cochran's heterogeneity p value was obtained.

**Supplementary Table 4.** Associations of Burkitt lymphoma (BL) with three *a priori*-specified HLA alleles in EMBLEM Uganda

| HLA Allele | Frequency | | OR [a] | *P* [a] |
|---|---|---|---|---|
| | Controls | Cases | | |
| A*02 | 0.18 | 0.12 | 0.61 | 0.001 |
| B*41 | 0.03 | 0.02 | 0.45 | 0.037 |
| B*58 | 0.12 | 0.09 | 0.77 | 0.121 |

[a] Estimates were obtained from generalized linear mixed models (GLMMs), accounting for genetic relatedness and adjusting for sex, age (continuous), *Plasmodium falciparum* positivity, and population structure using the top 3 principal components (PCs). Association statistics were conducted by a fixed-effect meta-analysis across four countries. All statistical tests are 2-sided.

**Supplementary Table 5.** Top HLA alleles associated with Burkitt lymphoma (BL) in EMBLEM and Malawi

| **Locus** | **HLA allele** | **Info (R2)** | **Frequency** | | **Meta-analysis [a]** | | | **Condition on HLA-DQA1*04:01 [a]** | |
|---|---|---|---|---|---|---|---|---|---|
| | | | Controls | Cases | OR (95% CI) | $P_{meta}$ | $P_{het}$ | OR (95% CI) | $P_{meta}$ |
| HLA-DQA1 | 04 | 0.99 | 0.08 | 0.12 | 1.60 (1.31, 1.96) | $5.02\times10^{-6}$ | 0.92 | n/a | n/a |
| HLA-DQB1 | 04 | 0.97 | 0.07 | 0.11 | 1.60 (1.30, 1.98) | $1.29\times10^{-5}$ | 0.87 | 1.06 (0.62, 1.82) | 0.83 |
| HLA-DQB1 | 04:02 | 1.00 | 0.07 | 0.11 | 1.61 (1.30, 1.99) | $1.16\times10^{-5}$ | 0.87 | 1.06 (0.62, 1.83) | 0.83 |
| HLA-DRB1 | 03:02 | 0.99 | 0.09 | 0.11 | 1.47 (1.20, 1.81) | $2.30\times10^{-4}$ | 0.49 | 1.04 (0.76, 1.43) | 0.81 |

[a] Estimates were obtained from generalized linear mixed models (GLMMs), accounting for genetic relatedness and adjusting for sex, age (continuous), *Plasmodium falciparum* positivity, and population structure using the top 3 principal components (PCs). Association statistics were conducted by a fixed-effect meta-analysis across four countries. Cochran's heterogeneity p value was obtained. All statistical tests are 2-sided.

**Supplementary Table 6.** Country-specific associations with Burkitt lymphoma (BL) with identified top HLA allele, Haplotype, GWAS loci [a]

| Locus | Variant | Info [b] | Eff. Allele/Ref. Allele [c] | Country | Frequency [d] | | Meta-analysis [e] |
|---|---|---|---|---|---|---|---|
| | | | | | Controls | Cases | OR (95% CI) |
| HLA-DQA1 | 04:01 | 0.99 | -- | Uganda | 0.06 | 0.08 | 1.48 (1.02, 2.17) |
| | | | | Kenya | 0.13 | 0.15 | 1.61 (1.15, 2.26) |
| | | | | Tanzania | 0.08 | 0.13 | 1.83 (1.16, 2.90) |
| | | | | Malawi | 0.08 | 0.12 | 1.59 (0.96, 2.63) |
| Class II haplotype | DRB1*03:02-DQA1*04:01-DQB1*04:02 | n/a | -- | Uganda | 0.04 | 0.07 | 1.51 (0.95, 2.38) |
| | | | | Kenya | 0.11 | 0.12 | 1.46 (1.00, 2.14) |
| | | | | Tanzania | 0.06 | 0.11 | 1.94 (1.18, 3.20) |
| | | | | Malawi | 0.06 | 0.1 | 1.54 (0.87, 2.72) |
| HLA-DQA1 | rs2040406 | 0.98 | G/A | Uganda | 0.26 | 0.34 | 1.41 (1.14, 1.74) |
| | | | | Kenya | 0.34 | 0.38 | 1.39 (1.08, 1.79) |
| | | | | Tanzania | 0.28 | 0.39 | 1.64 (1.20, 2.24) |
| | | | | Malawi | 0.29 | 0.36 | 1.38 (1.02, 1.85) |
| HLA-DQA1 | Gln-53 | 0.98 | -- | Uganda | 0.3 | 0.37 | 1.32 (1.07, 1.62) |
| | | | | Kenya | 0.36 | 0.4 | 1.28 (1.00, 1.64) |
| | | | | Tanzania | 0.3 | 0.41 | 1.66 (1.21, 2.27) |
| | | | | Malawi | 0.29 | 0.36 | 1.34 (1.00, 1.80) |

Abbreviations: HLA: human leukocyte antigen, OR: odds ratio, CI: confidence interval. All statistical tests are 2-sided.

a. Estimates were obtained from generalized linear mixed models (GLMMs), accounting for genetic relatedness and adjusting for sex, age (continuous), *falciparum* positivity, and population structure using the top 3 principal components (PCs).

b. Imputation Info ($R^2$) obtained from SNP2HLA.

c. Effect allele/reference allele; P stands for presence and A stands for absence for amino acid polymorphisms and HLA alleles.

d. For SNP rs2040406, the numbers indicate frequencies for effect allele.

e. Association statistics were conducted by a fixed-effect meta-analysis across four countries.

**Supplementary Table 7.** Sensitivity analyses 2-5 of associations of Burkitt lymphoma (BL) with *HLA-DQA1*04:01* in EMBLEM and Malawi studies [a]

| HLA allele | Sensitivity analysis 2 OR (95% CI) [b] | Sensitivity analysis 3 OR (95% CI) [c] | Sensitivity analysis 4 OR (95% CI) [d] | Sensitivity analysis 5 OR (95% CI) [e] |
|---|---|---|---|---|
| *HLA-DQA1*04:01* | 1.74 (1.37, 2.20) | 1.76 (1.38, 2.25) | 1.64 (1.32, 2.04) | 1.63 (1.31, 2.02) |

a Estimates were obtained from generalized linear mixed models (GLMMs) adjusting for sex, age (continuous), *P. falciparum* positivity, and population substructure using the top 3 country-specific principal components (PCs) and genetic relatedness. ORs and 95% CI were obtained based on a fixed-effect meta-analysis across four countries. All statistical tests are 2-sided.

b Models were additionally adjusted for residential area (rural vs. urban). Analysis was conducted restricting to EMBLEM study since information on residential area for subjects in Malawi was not available

c Models were additionally adjusted for recruitment season (wet vs. dry). Analysis was conducted restricting to EMBLEM study since information on residential area for subjects in Malawi was not available

d Models were additionally adjusted for two malaria susceptibility SNPs (*HBB*-rs334 and *ABO*-rs8176703).
e Excluded 14 subjects that were ancestry outliers in the PCA (3 in Kenya, 2 in Tanzania, and 9 in Malawi and Mozambique)

**Supplementary Table 8.** Association of Burkitt lymphoma (BL) with top GWAS loci ($P<1.1\times10^{-6}$) in the HLA region in EBMLEM and Malawi studies

| Variant | Position (bp) | Info (R2) | Eff. Allele | Ref. Allele | Frequency | | Meta-analysis [a] | | | Condition on rs2040406 | | ANNOVAR annotation | |
|---|---|---|---|---|---|---|---|---|---|---|---|---|---|
| | | | | | Controls | Cases | OR | *P* | $P_{het}$ | OR | *P* | Variant type | Nearby gene (distance or change) |
| rs1064994 | 32611195 | 0.98872 | C | T | 0.30 | 0.37 | 1.38 | $8.75\times10^{-7}$ | 0.67 | 0.98 | 0.91 | UTR3 | HLA-DQA1(NM_002122:c.*487 C>T) |
| rs1065049 | 32611275 | 0.98621 | A | C | 0.30 | 0.37 | 1.39 | $6.06\times10^{-7}$ | 0.66 | 1.02 | 0.91 | UTR3 | HLA-DQA1(NM_002122:c.*567 A>C) |
| rs9272982 | 32611376 | 0.98342 | A | G | 0.31 | 0.38 | 1.38 | $8.23\times10^{-7}$ | 0.72 | 0.90 | 0.60 | UTR3 | HLA-DQA1(NM_002122:c.*668 A>G) |
| rs1130399 | 32629755 | 0.99011 | A | G | 0.19 | 0.26 | 1.48 | $1.21\times10^{-7}$ | 0.78 | 1.26 | 0.01 | exonic | HLA-DQB1 |

a Estimates were obtained from generalized linear mixed models (GLMMs), accounting for genetic relatedness and adjusting for sex, age (continuous), falciparum positivity, and population structure using the top 3 principal components (PCs) by country. ORs were obtained based on a fixed-effect meta-analysis across four countries. Cochran’s heterogeneity p value was obtained. All statistical tests are 2-sided.

**Supplementary Table 9.** Association of Burkitt lymphoma (BL) with top loci with previously reported pleiotropic associations with anti-EBV IgG

| Trait | Variant | Position (bp) | Nearby gene | Eff. Allele/Ref. Allele | *P* for anti-EBV IgG [a] | Frequency | | Meta-analysis [b] | | |
|---|---|---|---|---|---|---|---|---|---|---|
| | | | | | | Controls | Cases | OR | P | $P_{het}$ |
| anti-EBNA1 IgG | rs1064991 | 32611176 | HLA-DQA1 | C/G | $8.25\times10^{-17}$ | 0.31 | 0.38 | 1.36 | $2.11\times10^{-6}$ | 0.61 |
| anti-EBNA1 IgG | rs3129867 | 32404220 | HLA-DRA | G/C | $2.10\times10^{-24}$ | 0.43 | 0.41 | 0.84 | $8.24\times10^{-3}$ | 0.60 |
| anti-EBNA1 IgG | rs6927022 | 32612397 | HLA-DRB1 | G/A | $1.36\times10^{-21}$ | 0.26 | 0.31 | 1.18 | $1.90\times10^{-2}$ | 0.21 |
| anti-VCA IgG | rs28394498 | 32615738 | HLA-DQA1 | T/A | $1.57\times10^{-12}$ | 0.21 | 0.19 | 0.91 | 0.256 | 0.93 |

a adapted from Sallah et al. *Nat Commun* 2020 (ref. #28)

[b] Estimates were obtained from generalized linear mixed models (GLMMs), accounting for genetic relatedness and adjusting for sex, age (continuous), falciparum positivity, and population structure using the top 3 principal components (PCs) by country. ORs were obtained based on a fixed-effect meta-analysis across four countries. Cochran's heterogeneity p value was obtained. All statistical tests are 2-sided.

**Supplementary Table 10.** Table showing functional annotation for locus rs2040406 using several public databases

| SNP | Annovar annotation | RegulomeB D score [a] | Haploreg | GWAS Catalog | GTExv8 |
|---|---|---|---|---|---|
| rs2040406 | Intergenic | 5: minimal binding evidence | significant eQTL hit | reported in multiple sclerosis ($p=1\times10^{-20}$) [b] | significant eQTL HLA-DQA1/C4A |

a The RegulomeB D scoring refers to the DNA features and regulatory regions evidence being 1a the highest level of evidence and 6 the lowest.

b. PMID: 20453840

**Supplementary Table 11.** Association between Burkitt lymphoma (BL) and common HLA alleles (allele frequency>5%; reference: rare alleles) in EMBLEM and Malawi.

| Locus | HLA gene | Common alleles Frequency | | Meta-analysis [a] | | |
|---|---|---|---|---|---|---|
| | | Controls | Cases | OR | $P$ | $P_{het}$ |
| **HLA class I gene** | | | | | | |
| | A | 0.55 | 0.53 | 0.86 | 0.016 | 0.39 |
| | B | 0.47 | 0.47 | 0.99 | 0.822 | 0.37 |
| | C | 0.69 | 0.69 | 0.92 | 0.238 | 0.68 |
| **HLA class II gene** | | | | | | |
| | DPB1 | 0.59 | 0.56 | 1.04 | 0.576 | 0.89 |
| | DQB1 | 0.91 | 0.89 | 0.85 | 0.144 | 0.32 |
| | DRB1 | 0.69 | 0.67 | 0.99 | 0.854 | 0.22 |

[a] Estimates were obtained from generalized linear mixed models (GLMMs), accounting for genetic relatedness and adjusting for sex, age (continuous), falciparum positivity, and population structure using the top 3 principal components (PCs) by country. ORs were obtained based on a fixed-effect meta-analysis across four countries. Cochran's heterogeneity p value was obtained. All statistical tests are 2-sided.

**Supplementary Table 12.** Association between Burkitt lymphoma (BL) and HLA zygosity in EMBLEM and Malawi

| HLA | Locus | Frequency of homozygosity | | Meta-analysis [a] | | |
|---|---|---|---|---|---|---|
| | | Cases | Controls | OR | *P* | $P_{het}$ |
| **HLA class I gene** | | | | | | |
| | A | 0.05 | 0.07 | 0.70 | 0.069 | 0.363 |
| | B | 0.04 | 0.06 | 0.64 | 0.044 | 0.762 |
| | C | 0.07 | 0.11 | 0.62 | 0.004 | 0.782 |
| **HLA class II gene** | | | | | | |
| | DPB1 | 0.19 | 0.18 | 1.16 | 0.191 | 0.668 |
| | DQB1 | 0.15 | 0.19 | 0.83 | 0.120 | 0.628 |
| | DRB1 | 0.09 | 0.10 | 1.10 | 0.556 | 0.743 |

[a] Estimates were obtained from generalized linear mixed models (GLMMs), accounting for genetic relatedness and adjusting for sex, age (continuous), falciparum positivity, and population structure using the top 3 principal components (PCs) by country. ORs were obtained based on a fixed-effect meta-analysis across four countries. Cochran's heterogeneity p value was obtained. All statistical tests are 2-sided.
