# Supplementary material for: Human leukocyte antigen-DQA1*04:01 and rs2040406 variants are associated with elevated risk of childhood Burkitt lymphoma
Source: Commun Biol. 2024 Jan 5;7:41. doi: 10.1038/s42003-023-05701-5 (PMC10770398; doi:10.1038/s42003-023-05701-5)
Supplement: Supplementary file 3 — Description of Additional Supplementary Files [file 42003_2023_5701_MOESM3_ESM.pdf]

Description of Additional Supplementary Files

File name: Supplementary Data 1

Description: The source data behind the graphs in Figure 2.
