# Supplementary material for: Human leukocyte antigen-DQA1*04:01 and rs2040406 variants are associated with elevated risk of childhood Burkitt lymphoma
Source: Commun Biol. 2024 Jan 5;7:41. doi: 10.1038/s42003-023-05701-5 (PMC10770398; doi:10.1038/s42003-023-05701-5)
Supplement: Supplementary file 5 — Reporting Summary [file 42003_2023_5701_MOESM5_ESM.pdf]

nature portfolio

Corresponding author(s): Sam M. Mbulaiteye

Last updated by author(s): Nov 27, 2023

Reporting Summary

Nature Portfolio wishes to improve the reproducibility of the work that we publish. This form provides structure for consistency and transparency in reporting. For further information on Nature Portfolio policies, see our Editorial Policies and the Editorial Policy Checklist.

Statistics

For all statistical analyses, confirm that the following items are present in the figure legend, table legend, main text, or Methods section.

n/a	Confirmed	
☐	☒	The exact sample size (*n*) for each experimental group/condition, given as a discrete number and unit of measurement
☐	☒	A statement on whether measurements were taken from distinct samples or whether the same sample was measured repeatedly
☐	☒	The statistical test(s) used AND whether they are one- or two-sided<br>*Only common tests should be described solely by name; describe more complex techniques in the Methods section.*
☐	☒	A description of all covariates tested
☐	☒	A description of any assumptions or corrections, such as tests of normality and adjustment for multiple comparisons
☐	☒	A full description of the statistical parameters including central tendency (e.g. means) or other basic estimates (e.g. regression coefficient) AND variation (e.g. standard deviation) or associated estimates of uncertainty (e.g. confidence intervals)
☐	☒	For null hypothesis testing, the test statistic (e.g. *F*, *t*, *r*) with confidence intervals, effect sizes, degrees of freedom and *P* value noted<br>*Give P values as exact values whenever suitable.*
☒	☐	For Bayesian analysis, information on the choice of priors and Markov chain Monte Carlo settings
☒	☐	For hierarchical and complex designs, identification of the appropriate level for tests and full reporting of outcomes
☐	☒	Estimates of effect sizes (e.g. Cohen's *d*, Pearson's *r*), indicating how they were calculated

Our web collection on statistics for biologists contains articles on many of the points above.

Software and code

Policy information about availability of computer code

Data collection	Questionnaire data for EMBLEM was processed using DataFax under the NIAID Office of Cyber Infrastructure and Computational Biology clinical research support program. No other software was used for data collection.
Data analysis	1. Association analysis were conducted using generalized linear mixed models (GLMMs) with the logit link in Biowulf.<br>2. Principal components (PC) analysis was done using 787,731 uncorrelated SNPs (at r2 < 0·3) in the genotyped data to generate population-specific PCs used to control for ancestry. The top 3 population-specific PCs were used to adjust for ancestry.<br>3. We used NAToRA, a relatedness-pruning method to minimize the loss of dataset size in genetic and omics analyses, to construct relationship matrix to use control for relatedness in the dataset .<br>4. We adjusted for multiple comparisons using a Bonferroni correction based on the effective degrees of freedom estimated from the number of uncorrelated loci in each gene based on the number of effective tests performed after considering correlated SNPs within the gene, i.e., 0·05/number of effective tests.

For manuscripts utilizing custom algorithms or software that are central to the research but not yet described in published literature, software must be made available to editors and reviewers. We strongly encourage code deposition in a community repository (e.g. GitHub). See the Nature Portfolio guidelines for submitting code & software for further information.

Data

Policy information about availability of data

All manuscripts must include a data availability statement. This statement should provide the following information, where applicable:

- Accession codes, unique identifiers, or web links for publicly available datasets
- A description of any restrictions on data availability
- For clinical datasets or third party data, please ensure that the statement adheres to our policy

The genome-wide and phenotypic data from the Ghana Prostate Healthy Study and the EMBLEM and Malawi studies are publicly available. The previously published data from the Ghana Prostate Healthy Study are available under restricted access in the Genomic Data Commons; access to these studies is available through dbGaP: Ghana Prostate Study (phs000838.v1.p1 [https://www.ncbi.nlm.nih.gov/projects/gap/cgi-bin/study.cgi?study_id=phs000838.v1.p1]). The genetic data of participants in the EMBLEM and Malawi studies are under controlled access (requires IRB approval and are limited to not-for-profit research). Readers can access the data by applying via dbGaP under accession link phs001705.v1.p1 [https://www.ncbi.nlm.nih.gov/projects/gap/cgi-bin/study.cgi?study_id=phs001705.v1.p1]. The remaining data reported in the article are available within the Article, Supplementary Information, or Source Data file provided with this paper.

Research involving human participants, their data, or biological material

Policy information about studies with human participants or human data. See also policy information about sex, gender (identity/presentation), and sexual orientation and race, ethnicity and racism.

Reporting on sex and gender	Results are controlled for sex to adjust for the the biologic effects of sex on Burkitt lymphoma. Sex was collected on questionnaire and then confirmed by genetic testing based on the identifier. When reported sex was discordant with genetic sex (in 31 instances), the genetic sex was considered the sex of that individual. We made decision after reviewing multiple forms where sex was recorded, usually by different observers who recorded the sex from the guardians, and noting that sex reported on these forms was usually consistent. Karyotypes of some of the discordant individuals revealed abnormalities such as XO, XXY, which suggested to us that some children had ambiguous sex , but parents in villages usually assign sex and use it consistently.
Reporting on race, ethnicity, or other socially relevant groupings	Results are controlled for population structure using top 3 principal components.
Population characteristics	The study includes 4, 645 children (800 with Burkitt lymphoma and 3, 845 controls) aged 0-15 years enrolled in Uganda, Tanzania, Kenya, and Malawi. Most participants were from the EMBLEM study in Uganda, Tanzania, and Kenya (71.9% of the cases and 94.5% of controls. As expected and by study design, compared to controls, BL cases were predominantly male (62.9% vs. 52.1%) and were aged 3-11 years old (77.9% of the cases vs. 74.5% of the controls). P. falciparum infection was detected in 282 (35.3%) of BL cases vs. 1857 (48.3%) controls.
Recruitment	The participants are from two studies: a) The EMBLEM study was a population-based case-control study that enrolled participants from six regions, two neighboring in Uganda along the River Nile in northern Uganda and four neighboring in Tanzania and Kenya on the southern shores of Lake Victoria during 2010-2016. The BL cases were diagnosed at participating hospitals, and diagnosed by histology or cytology (61% of cases) and compatible clinical and laboratory investigation in the remainder. Controls were apparently healthy children enrolled from 300 villages randomly selected in the study region. The controls were enrolled either in their village as matched controls (selected based on age and sex distribution of BL cases) or as survey controls, where all children in the selected village were enrolled, or as healthy facility controls for children attending village health facilities for minor ailments. The main biases for cases was incomplete ascertainment of cases, while for controls, the main concern was representativeness of the controls for children in the study area.<br>b) The Malawi study was hospital-based conducted at a tertiary-level hospital where all children were attending because of suspected cancer during 2005-2008. This is a case-case study where children with BL were compared to children with other cancers. BL diagnosis was based on local histology or cytology, or compatible clinical and laboratory investigation. Children with other solid tumors were used as controls. Those with Kaposi sarcoma, HIV positivity, and lymphoid or leukemic diagnoses were excluded from the controls.
Ethics oversight	We confirm that all relevant ethical regulations were followed. Specifically, approval for the EMBLEM study was granted by ethics committees at the Uganda Virus Research Institute (GC/127), Uganda National Council for Science and Technology (H816), Tanzania National Institute for Medical Research (NIMR/HQ/R.8c/Vol. IX/1023), Moi University/Moi Teaching and Referral Hospital (000536), and National Cancer Institute (10-C-N133). Ethical approval for the original Infections and Childhood Cancer Study was granted by ethics committees at the Malawi College of Medicine (P.03/04/277R) and Oxford University. Because the original Malawi Infections and Childhood Cancer study did not request participants to consent to genetic testing, special ethical approval to conduct genetic testing was obtained from the Malawi National Health Sciences Research Committee (Approval #2405). Written informed consent was obtained from participants' guardians in EMBLEM and Malawi studies, and written informed assent was obtained from children aged ≥7 years old in the EMBLEM study. The ethical approval for genetic studies favored research that would enable possible or suitable interventions in the communities where participants were enrolled or increasing knowledge considered relevant to the local communities, such as research on HLA variation and malaria resistance to investigate the association of BL with malaria.

Note that full information on the approval of the study protocol must also be provided in the manuscript.

Field-specific reporting

Please select the one below that is the best fit for your research. If you are not sure, read the appropriate sections before making your selection.

☒ Life sciences ☐ Behavioural & social sciences ☐ Ecological, evolutionary & environmental sciences

For a reference copy of the document with all sections, see nature.com/documents/nr-reporting-summary-flat.pdf

Life sciences study design

All studies must disclose on these points even when the disclosure is negative.

Sample size	The original study design was designed to have 80% statistical power to detect a minimum OR of 0·37-0·70 for BL for any loci in a study sample with 600 BL cases and 1500 controls, assuming MAFs of 0·05-0·5 and two-sided alpha = 0·05.
Data exclusions	The study considered 5,499 enrolled participants, 655 were excluded due to lack of sample suitable doe DNA extraction. Of 4,811 participants with suitable DNA , 105 were excluded (37 for insufficient DNA, 35 for failing DNA staging procedures, 13 for sample contamination, 3 for multiple aliquots, 5 for incomplete information, 5 for discordant gender, 1 doe DNA depletion, 3 for replicate samples, and 3 as twins) leaving a sample set of 4,739 2who were genotyped. Following genotyping, 94 subjects were excluded to achieve an analystic sample size of 4,645 (800 BL cases and 3, 845 controls).
Replication	The findings made here have not been replicated in another dataset because there are no datasets with suitable data and samples and permission for genetic testing. The results presented here provide new data and design features that may be used by other investigators to replicate the findings in other regions of Africa where malaria and BL co-occur.
Randomization	The study is descriptive, thus the randomization was not done.
Blinding	Genetic testing was done with lab staff blinded about the case-control status of the samples, as well as the other characteristics of the samples.

Reporting for specific materials, systems and methods

We require information from authors about some types of materials, experimental systems and methods used in many studies. Here, indicate whether each material, system or method listed is relevant to your study. If you are not sure if a list item applies to your research, read the appropriate section before selecting a response.

Materials & experimental systems

n/a	Involved in the study	
☒	☐	Antibodies
☒	☐	Eukaryotic cell lines
☒	☐	Palaeontology and archaeology
☒	☐	Animals and other organisms
☐	☒	Clinical data
☒	☐	Dual use research of concern
☒	☐	Plants

Methods

n/a	Involved in the study	
☒	☐	ChIP-seq
☒	☐	Flow cytometry
☒	☐	MRI-based neuroimaging

Clinical data

Policy information about clinical studies

All manuscripts should comply with the ICMJE guidelines for publication of clinical research and a completed CONSORT checklist must be included with all submissions.

Clinical trial registration	NCT01196520
Study protocol	The EMBLEM study protocol can be accessed at: https://emblem.cancer.gov/resources/index.html. The BLGSP protocol can be accessed at: https://ocg.cancer.gov/programs/cgci/projects/burkitt-lymphoma.
Data collection	Data collection in EMBLEM was conducted in six regions (2 neighboring in northern Uganda on opposite sides of the R. Nile and four neighboring in Tanzania and Kenya on the southern shores of L. Victoria) during 2010-2016. BL cases were enrolled at participating hospitals, while the controls were enrolled from 300 villages randomly selected from the six regions. The Malawi cases were enrolled at the Queen Elizabeth Hospital in Blantyre during 2005-2008. All children were referred for suspected cancer. BL cases were defined based on histological or cytological diagnosis with clinical and supportive laboratory results. Venous blood was collected before cancer treatment.
Outcomes	Burkitt lymphoma is the primary outcome of the case-control analysis. BL was diagnosed using local cytology and/or histology. In a proportion of cases, cytology or histology were not obtained, but the clinical, imaging, and laboratory results were compatible with a

diagnosis of BL. All controls did not have Burkitt lymphoma. In Malawi, the controls were comprised of children with other cancers.

P. falciparum infection was diagnosed using blood smears, rapid diagnostic tests in the EMBLEM study or PCR in Malawi.
